# Berry Cell Vitality Assessment and the Effect on Wine Sensory Traits Based on Chemical Fingerprinting, Canopy Architecture and Machine Learning Modelling

**DOI:** 10.3390/s21217312

**Published:** 2021-11-03

**Authors:** Sigfredo Fuentes, Claudia Gonzalez Viejo, Chelsea Hall, Yidan Tang, Eden Tongson

**Affiliations:** Digital Agriculture Food and Wine Group, School of Agriculture and Food, Faculty of Veterinary and Agricultural Sciences, University of Melbourne, Parkville, VIC 3010, Australia; cgonzalez2@unimelb.edu.au (C.G.V.); chall3@student.unimelb.edu.au (C.H.); yidant1@student.unimelb.edu.au (Y.T.); eden.tongson@unimelb.edu.au (E.T.)

**Keywords:** near-infrared spectroscopy, computer vision, sensory analysis, machine learning, berry cell death

## Abstract

Berry cell death assessment can become one of the most objective parameters to assess important berry quality traits, such as aroma profiles that can be passed to the wine in the winemaking process. At the moment, the only practical tool to assess berry cell death in the field is using portable near-infrared spectroscopy (NIR) and machine learning (ML) models. This research tested the NIR and ML approach and developed supervised regression ML models using Shiraz and Chardonnay berries and wines from a vineyard located in Yarra Valley, Victoria, Australia. An ML model was developed using NIR measurements from intact berries as inputs to estimate berry cell death (BCD), living tissue (LT) (Model 1). Furthermore, canopy architecture parameters obtained from cover photography of grapevine canopies and computer vision analysis were also tested as inputs to develop ML models to assess BCD and LT (Model 2) and the intensity of sensory descriptors based on visual and aroma profiles of wines for Chardonnay (Model 3) and Shiraz (Model 4). The results showed high accuracy and performance of models developed based on correlation coefficient (R) and slope (b) (M1: R = 0.87; b = 0.82; M2: R = 0.98; b = 0.93; M3: R = 0.99; b = 0.99; M4: R = 0.99; b = 1.00). Models developed based on canopy architecture, and computer vision can be used to automatically estimate the vigor and berry and wine quality traits using proximal remote sensing and with visible cameras as the payload of unmanned aerial vehicles (UAV).

## 1. Introduction

In grapevines, berry cell death (BCD) occurs from 90–100 days after anthesis within the mesocarp tissue of berries ([1]. It has been proposed that this process may be linked to an evolutionary trait to improve seed spreading [2]. However, it also has implications for winemaking since, when the berry mesocarp cells die, internal cellular compounds from different compartments may mix (vacuole and cytoplasm), potentially producing desirable flavors and aromas, among other processes, which are passed to the final wine [3]. Flavor and aroma are critical quality traits for berries and wine, along with different characteristics, such as berry softening, to increase the efficiency of berry crushing [4,5]. Previous research has shown that different grapevine cultivars present different patterns of BCD, and the final percentage is associated with levels of berry shriveling, which may be related to the development of specific characteristics or styles of the final wines [3,6]. Hence, monitoring BCD could be a novel tool to assess the effects of management practices and the environment on berry maturity and wine quality. On the contrary, for table grapes, the final BCD is minimal or non-existent, which may be related to artificial selection processes of their different cultivars for fresh consumption, with required berry characteristics, such as high juiciness, fruity aromas, sweet flavor, crunchiness, and turgidity, among others [1,3].

Environmental factors are also linked to changes in the slope of BCD, such as higher ambient temperatures [7] and water deficits [5], which can produce steep seasonal BCD slopes, which can be exacerbated as a direct result of climate change forecasts [8]. Furthermore, microclimatic characteristics at the fruit zone level, given by canopy vigor, structure, and architecture, may also play a role in cell death rates throughout the season [9].

Assessing BCD currently involves time-consuming berry collections and specialized personnel for laboratory analysis using fluorescent stains of the mesocarp and microscopy imaging coupled with computer vision algorithms to obtain percentages of BCD and living tissue (LT) [6]. Sensor technology involving impedance measurements through the berries linked to BCD has been proposed to make the assessment more expedited and practical [10]. However, these measurements were performed in the laboratory without a proposed portable device for in-field assessment capabilities, making it less practical and able to measure sentinel berries on specific vines only. On the other hand, recent research has focused on the non-destructive measurements of berries using portable near-infrared (NIR) spectrometers to estimate BCD and LT in Pinot Noir grapes using machine learning modeling techniques [3]. From the latest research, accurate models were developed with NIR readings within the 1600–2500 nm range that included some of the signal (overtones) related to hydrogen peroxide (H_2_O_2_ between 1420–1650; [11]), which can be directly linked to the BCD process [12].

This paper aimed to develop machine learning (ML) models based on NIR spectroscopy and canopy architecture parameters as inputs to predict the BCD, LT of berries, and potential wine acceptability by consumers for two of the most important cultivars in Australia: Shiraz and Chardonnay. Specifically, ML Model 1 was developed using the NIR absorbance values of berry samples (1596–1919 nm; Model 1) and canopy architecture parameters (Model 2) as inputs to predict living (LT) and dead tissue (BCD). On the other hand, Models 3 and 4 were constructed using canopy architecture parameters, obtained from upward pictures of canopies and computer vision algorithms as inputs to predict the intensity of sensory descriptors for Chardonnay (Model 3) and Shiraz (Model 4).

Implementing these models close to harvest to predict the BCD, LT, and final wine quality traits may offer a more comprehensive berry maturity assessment to target the specific wine quality or style that characterizes a particular region. The assessment of canopy architecture parameters required for these models can be obtained from smartphone applications such as VitiCanopy [13] or aerial and high-resolution satellite imagery [14]. Furthermore, by relating canopy architecture parameters with berry quality, it may be possible to target canopy management practices to obtain more uniform berry quality traits within the vineyards.

## 2. Materials and Methods

### 2.1. Site and Grapevine Cultivars Description

**Figure 1:**
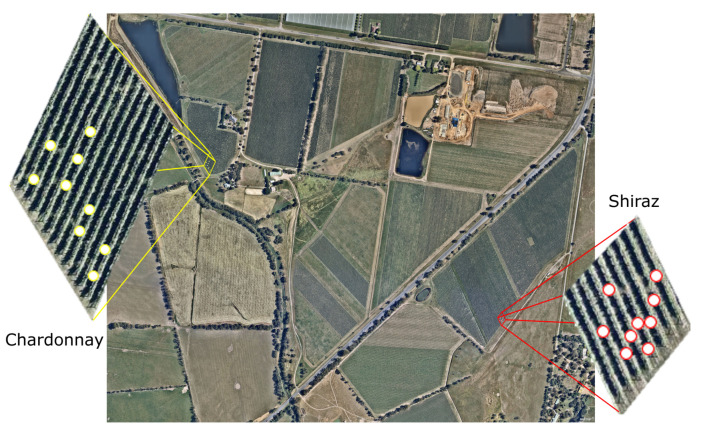
Location of trial sites and blocks for Chardonnay (yellow magnification) and Shiraz (red magnification) and respective vines monitored.

Samples were collected from the Coombe vineyard in Coldstream, Victoria, Australia (−37°41′42.468″ S, 145°24′31.068″ E) at an elevation of 83 m. The commercial vineyard is located in the Yarra Valley with a total extension of 49 Has. The region is characterized by an oceanic climate with an average maximum temperature of 20.5 °C and a mean January temperature (MJT) of 28.1 °C with an average annual rainfall of 732.3 mm (Bureau of Meteorology, Australia). Samples were obtained from two different cultivars: Chardonnay, clone Schwarzmann from Block B and Shiraz clone R99 in Block F (Figure 1), which used the vertical shoot positioning (VSP) training system. Berry and canopy data from Chardonnay vines were gathered on two different dates towards the end of the season at 98 days after anthesis (DAA98) and 101 days after anthesis (DAA101). On the other hand, for Shiraz, data were obtained on three different dates at 94 days after anthesis (DAA94), 97 days after anthesis (DAA97), and 108 days after anthesis (DAA108). To obtain the variability required for modeling purposes, three plants from a low, medium and high canopy vigor based on the leaf area index (LAI) measured using the VitiCanopy App [13] were selected and marked per cultivar (*n* = 9 plants per cultivar; 18 in total) (Figure 1).

### 2.2. Canopy Imaging and Digital Measurements

**Figure 2:**
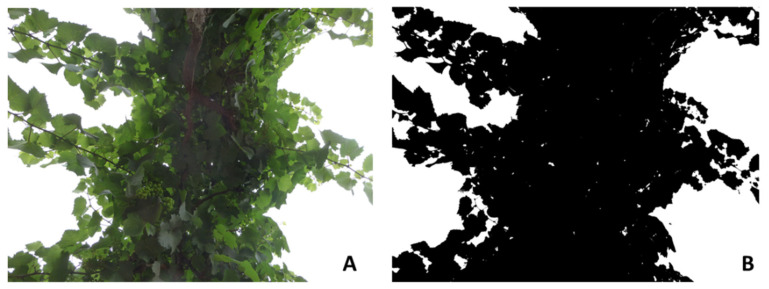
Example of (**A**) an upward-looking digital image from a grapevine canopy and (**B**) binarization for gap analysis to obtain canopy architecture parameters.

For canopy vigor analysis based on imaging, an iPhone 11 (Apple Inc., Cupertino, CA, USA; resolution: 12 megapixels) was mounted on a selfie stick. Two upward-looking images (Figure 2) at ground level were obtained at ~70–80 cm from each side of the canopy per plant (one at each side of the trunk) for nine grapevines per cultivar (*n* = 18 per cultivar; 36 total per date). Images were analyzed using a customized Matlab^®^ R2020b code [15,16] (Mathworks Inc., Natick, MA, USA), which is capable of obtaining canopy architecture parameters such as the LAI, effective LAI (LAI_e_), crown cover (*f_f_*), canopy cover (*f_c_*), crown porosity (Φ), and Clumping Index (Ω) as described in previous publications [3,13,14,16,17].

### 2.3. Near-Infrared Spectroscopy

Before undergoing the living/death tissue assessment described below, Intact berries were measured using a near-infrared spectroscopy device microPHAZIR™ RX Analyzer (Thermo Fisher Scientific, Waltham, MA, USA). This apparatus can measure within a spectral range between 1596 and 2396 nm. For each measurement, the whole berry was placed on the nose of the device, covering the entire reading area. Three NIR readings were taken per berry (*n* = 216 each cultivar), rotating the berry a third after each measurement. For data analysis, the first derivative based on Savitzky–Golay was calculated using Unscrambler X ver. 10.3 (CAMO Software, Oslo, Norway) software to enhance the peaks in the hydrogen peroxide (H_2_O_2_) range (1420–1650; [11]).

### 2.4. Living and Dead Tissue Analysis from Berries

From each of the nine plants marked of each cultivar, two bunches were marked from the east and west sides. From each bunch, six berries were collected from the middle of the bunches (*n* = 216). All berries collected were halved using a sharp blade longitudinally through the pericarp at the highest diameter. One half was used to measure total soluble solids (°Brix) using an Alla France REFBX010 optical refractometer (Alla France Sarl, Chemillé-Melay, France). The other half was used for staining with fluorescein diacetate (FDA) to acquire fluorescent microscopy images. The staining and fluorescence imaging followed the method described by Fuentes et al. [6]. The fluorescent microscope used for this measurement was a Leica DMC2900 (Leica, Wetzlar, Germany), and samples were viewed under a Leica M205 FA camera (Leica, Wetzlar, Germany).

**Figure 3:**
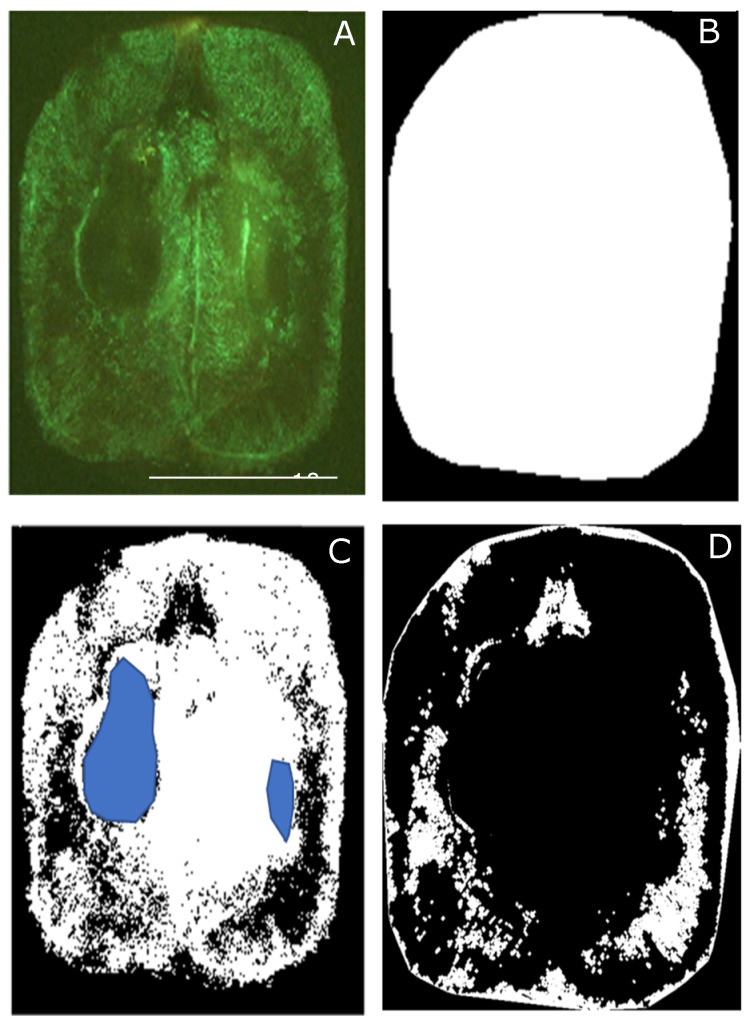
Example of fluorescent image (**A**) and analysis using computer vision algorithms for a Shiraz berry to obtain the berry contour and dimensions through edge recognition (**B**), living tissue, and seed extraction (**C**), and cell death quantification and patterns (**D**).

Images were obtained using the Leica Application Suite (LAS) software (Leica, Wetzlar, Germany) color cooled digital camera with the same gain and exposure settings for all images (Figure 3A). Berry images were analyzed using a code written in Matlab^®^ R2020b (Mathworks Inc., Natick, MA, USA) [3,6], which can recognize the berry shape automatically (Figure 3B). Binarized images were obtained, representing the living tissue (LT) within berries (Figure 3C, white). If seeds were seen in the image, which is surrounded by LT, a cropping selection was made manually to extract the seed to avoid over-estimating BCD (Figure 3C, blue region). Finally, a BCD binary image is obtained by inverting the LT binary image (Figure 3D). All berry images and BCD images were automatically saved as Joint Photographic Group (JPG) files.

### 2.5. Winemaking and Descriptive Sensory Analysis

Bunches of berries from each plant and cultivar were collected at harvest for micro-vinification. Three batches (referred as groups in results) of wine from each cultivar were made using berries from each group of plants, which were the same used for BCD and canopy architecture analysis. Wines were made in the servery area from the sensory laboratory at the Faculty of Veterinary and Agricultural Sciences (FVAS) from the University of Melbourne (UoM). For both cultivars, bunches were destemmed (~3–3.5 kg) and crushed, followed by their transfer to 5 L carboys. For Chardonnay, Saccharomyces cerevisiae EnartisFerm Vintage White yeast (0.25 g L^−1^; Enartis Pacific, Malvern East, VIC, Australia) was added to undergo fermentation, whilst for Shiraz, Saccharomyces cerevisiae EnartisFerm Vintage Red yeast was used (0.25 g L^−1^) for alcoholic fermentation, all batches were stored at 20 °C for 2 weeks.

For descriptive sensory analysis, a total of 11 trained panelists participated in the Quantitative Descriptive Analysis (QDA^®^) sensory session. Due to the lockdown in 2020 as a result of the COVID-19 pandemic, the sensory session was conducted remotely in the panelists’ residences. For this purpose, the wine samples were poured into 30 mL plastic test tubes, labelled with 3-digit random codes and mailed to the participants as express delivery in boxes with foam insulating material so that they received them within 1 day to avoid the wines from developing any off-aromas. For safety issues, panelists were asked to assess the visual and aroma characteristics of the wines only. Prior to the sessions, participants were asked to read and sign a consent form (Ethics ID: 1953926.4). Participants were asked to join a Zoom meeting (Zoom Video Communications, San Jose, CA, USA) and open a link to RedJade software (RedJade Sensory Solutions, LLC, Martinez, CA, USA) with the questionnaire. Participants were also asked to place the samples in front of them. The questionnaire consisted of assessing the intensity of visual parameters, aromas, and perceived quality (Table 1), which were rated using a 15 cm non-structured scale; both cultivars had a different questionnaire due to the distinct aromas they have.

### 2.6. Statistical Analysis and Machine Learning Modeling

Canopy architecture and sensory data were analyzed using ANOVA (*p* < 0.05) and the Tukey test (α = 0.05) in XLSTAT 2020.3.1 (Addinsoft, New York, NY, USA) to assess significant differences between samples. The mean and standard error (SE) values were also calculated for all parameters.

**Figure 4:**
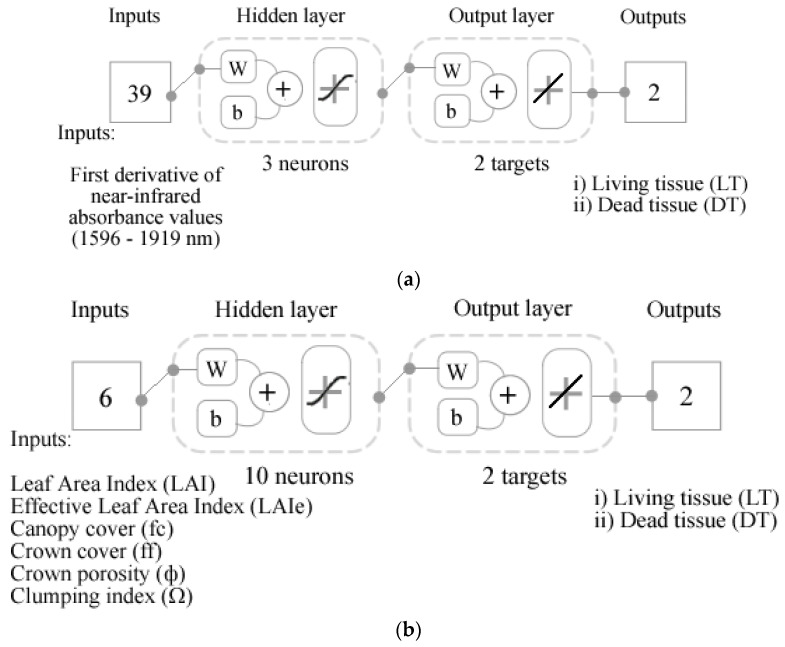

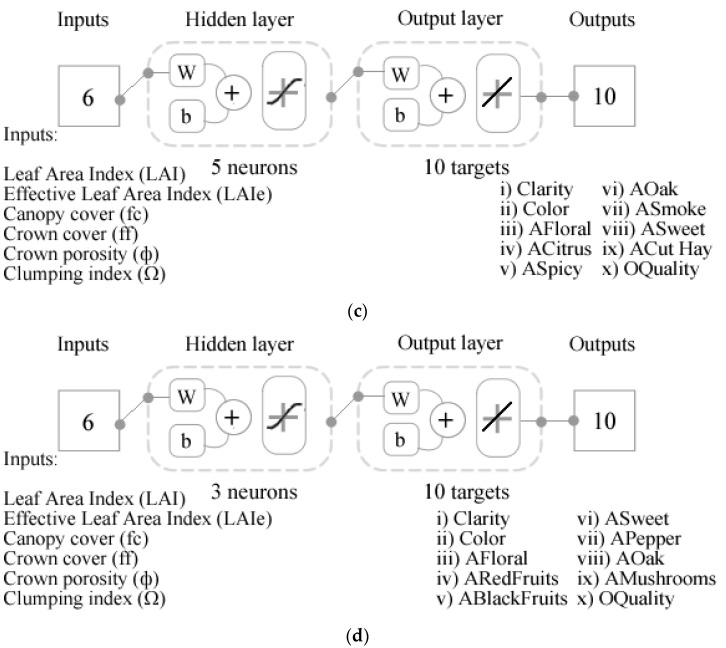
Diagrams representing the artificial neural network (ANN) two-layer feedforward models with a tan-sigmoid function in the hidden layer and a linear transfer function in the output layer. (**a**) Model 1 developed using the near-infrared absorbance values as inputs to predict living and dead tissue; (**b**) Model 2 developed using the canopy architecture parameters to predict living and dead tissue; (**c**) Model 3 using the canopy architecture parameters to predict the intensity of sensory descriptors of Chardonnay; (**d**) Model 4 using the canopy architecture parameters to predict the intensity of sensory descriptors of Shiraz. Abbreviations: W: weights; b: bias. Abbreviations from (**c**,**d**) are shown in Table 1.

Four ML regression models based on artificial neural networks (ANN) were developed using a code written in Matlab^®^ R2020b that automatically assesses 17 training algorithms to find the best model based on accuracy, slope, and performance. Hence, Model 1 was developed using the Bayesian Regularization algorithm with the first derivative of the NIR absorbance values (1596–1919 nm) as inputs to predict (i) living tissue (LT) and (ii) dead tissue (DT). Data were randomly divided, with 75% of the samples used for the training stage and 25% for testing using the mean squared error (MSE) as performance algorithm. Models 2–4 were developed using the Levenberg–Marquardt training algorithm. Model 2 was constructed using the canopy architecture parameters (i) LAI, (ii) LAIe, (iii) fc, (iv) ff, (v) φ, (vi) Ω as inputs to predict (i) LT and (ii) DT. On the other hand, Models 3 and 4 were developed using the canopy architecture variables as inputs to predict the intensity of sensory descriptors (Table 1) for Chardonnay (Model 3) and Shiraz (Model 4). For Models 2–4, the data were randomly divided as 60% of the samples for the training stage, 20% for validation with MSE performance algorithm, and 20% for testing. Neuron trimming was performed using 3, 5, 7, and 10 neurons to find the best and most efficient models with no under- or overfitting, obtaining the best models with three neurons for Models 1 and 4, ten neurons for Model 2, and five neurons for Model 3 (Figure 4). A diagram of the full process, including methods to obtain inputs, is shown in Supplementary Materials (Figure S1).

## 3. Results

For LT and LAIe at harvest, it was found that Chardonnay had lower LT and higher LAIe than Shiraz (Figure S2). According to the ANOVA, there were non-significant differences (*p* > 0.05) between the three groups of samples for LT in both cultivars. For LAIe, there were non-significant differences (*p* > 0.05) between the groups of samples for Chardonnay, but there were significant differences (*p* < 0.05) for Shiraz. For the latter, Group 3 (0.91) presented significant differences from Group 1 (1.44).

Table 2 shows the mean values, and ANOVA results from the canopy architecture parameters. It can be observed that for Chardonnay, there were non-significant differences (*p* > 0.05) between samples. For Shiraz, there were significant differences (*p* < 0.05) for all parameters except for Ω. Group 3 of Shiraz presented significantly higher LAI (2.00), fc (0.68), and ff (0.84) than Group 1 (LAI: 1.27; fc: 0.49; ff: 0.67). On the other hand, Group 3 was significantly lower in φ (0.19) than Group 1 (0.28) and 2 (0.21).

For the intensity of sensory descriptors of the three groups of Chardonnay and Shiraz wines, according to the ANOVA and Tukey test, there were non-significant (*p* > 0.05) differences between the groups of samples for any of the sensory attributes in wines from both cultivars (Table S1).

Table 3 shows that Model 1 had high accuracy based on the overall correlation coefficient (R = 0.87) to predict living and dead tissue using the NIR absorbance values from 1596 to 1919 nm. However, Model 2 presented higher overall accuracy (R = 0.98) when using canopy architecture parameters as inputs. For both models, the MSE values of training (Model 1: MSE = 35.2; Model 2: MSE = 8.9) were lower than testing (Model 1: MSE = 106.5; Model 2: MSE = 11.4), which is a sign of no under- or overfitting; furthermore, in Model 2, the validation and testing MSE values (MSE = 11.5 and 11.4, respectively) were similar, which is another sign of no under- or overfitting for Levenberg–Marquardt training algorithms.

**Figure 5:**
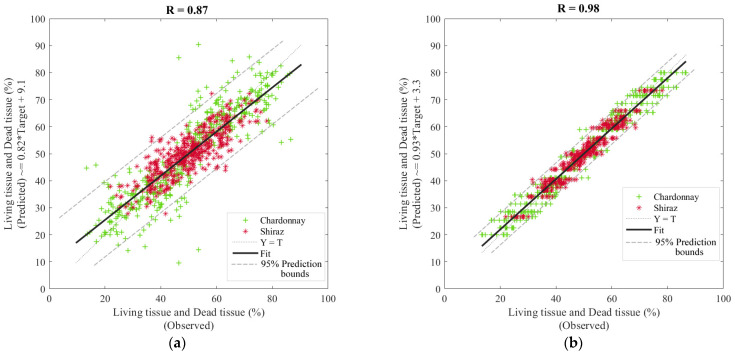
Overall artificial neural network regression models, where (**a**) depicts Model 1 developed using near-infrared absorbance values (1596–1919 nm) as inputs and (**b**) Model 2 developed using the canopy architecture parameters as inputs, both to predict living and dead tissue. Abbreviations: T: targets; R: correlation coefficient.

Figure 5 shows the overall regression models; it can be observed that Model 2 presented fewer outliers (4.17%; 36 out of 864) than Model 1 (4.98%; 43 out of 864) according to the 95% prediction bounds. Furthermore, most of the outliers in Model 1 were from Chardonnay samples.

Table 4 shows that both Models 3 (Chardonnay) and 4 (Shiraz) presented the same overall accuracy (R = 0.99) to predict the intensity of sensory ten descriptors (Table 1) using the canopy architecture parameters as inputs. Both models presented very high slopes (~1.00) and no signs of under- or overfitting as the training MSE values are lower than the testing, and the latter is the same as validation MSE.

**Figure 6:**
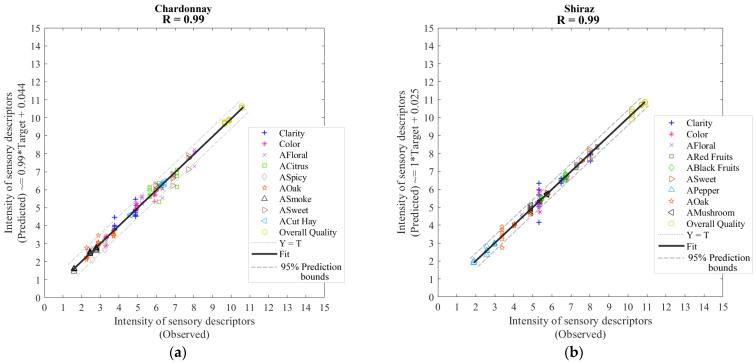
Overall artificial neural network regression models developed using the canopy architecture parameters as inputs to predict the intensity of sensory descriptors for (**a**) Chardonnay (Model 3) and (**b**) Shiraz (Model 4). Abbreviations: T: targets; R: correlation coefficient.

Figure 6 shows the overall regression models of Chardonnay (Figure 6a) and Shiraz (Figure 6b). According to the 95% prediction bounds, both models presented the same number of outliers (5%; 108 out of 2160).

## 4. Discussion

### 4.1. Dynamics of Berry Cell Death and Berry Composition

Berry dynamics in terms of BCD for Shiraz followed trends close to BCDs for the same cultivars reported earlier at harvest (Figure S2), which are also supported by previous studies on Shiraz grapevines from the Barossa Valley in South Australia [5,7]. It has also been shown that BCD inversely correlates with berry shrivel (positive correlation with LT) for Shiraz and other grapevine cultivars [6]. However, Chardonnay does not shrivel as much at harvest [5]. Furthermore, strong inverse correlations are expected between BCD and sugar concentration in berries (Brix); however, these two processes are not coupled, and they can be managed differentially using either canopy management strategies or cooling down the microclimate of bunches through shading [5] or misting [9]. The latter techniques have been investigated as methods to ameliorate the negative effects of climate change (mostly higher temperatures and lower rainfall) on berry shriveling and excessive sugar accumulation in berries, which lead to issues such as high alcohol content in wine [18,19,20].

### 4.2. Proximal Near-Infrared Spectroscopy of Berries and Machine Learning Modeling

Previous studies have shown that the light source used with the NIR spectrometer can penetrate the skin, pericarp, and part of the mesocarp up to around 3–4 mm in depth of intact berries [21]. The NIR instrument may measure through BCD and LT from any part of the edge of the berry. This capability supports the multitarget (BCD and LT) for machine learning modeling used in this study (Figure 5a).

Most of the NIR overtones related to sugar content in berries can be found between 650–1200 nm [22,23]. Considering that there is an apparent but not coupled relationship between the BCD and sugar content in berries, using NIR data linked to that particular NIR range may introduce bias into the ML developed targeting BCD. Hence, the latter NIR range should be avoided to prevent overfitting related to sugar-related artifacts in estimating BCD and LT [3]. For this research, ML models were tested using the Microphazir NIR range as an input to predict total soluble solids (TSS) and resulted in low accuracy (training: R = 0.46; validation: R: 0.46; testing: R = 0.05; overall: R = 0.39) and performance (training: MSE = 1.45; validation: MSE: 1.37; testing: MSE = 2.61), demonstrating the unlinked NIR range to TSS used to estimate BCD and LT. Within the range selected for ML modeling (1300–1830 nm), it also includes overtones for H_2_O_2_ concentrations [11] related to dead tissue in berries [12]. In this same range, other distinct overtones related to flavor and aroma can be found, which has the potential to model these parameters associated with BCD and LT, as demonstrated in previous studies [3].

### 4.3. Canopy Architecture, Berry Cell Vitality and Machine Learning Modeling

In grapevines, canopy architectures that achieve appropriate fruit and leaf exposure and a balance between the vegetative and reproductive organs allow growers to achieve the targeted quality traits of grapes and the resulting wines. Hence, it is necessary to have rapid, cost-effective, and accurate tools to monitor canopy architecture, potentially serving as a basis for modelling specific berry and wine quality traits. Cover photography has been applied successfully to monitor canopy architecture parameters for eucalyptus trees [24,25,26], apple trees [27], cherry trees [17,28] and grapevines [13,16,29,30]. From the latter studies, a computer application (App) was developed (VitiCanopy), which can use the camera and GPS capabilities of smartphones to obtain digital images and process them to obtain canopy architecture parameters [13]. The VitiCanopy App has been tested to assess vineyard variability [31], the effect of canopy management [29,30], and the effect on grape and wine quality [32]. Other research has found a direct impact of canopy architecture parameters in the aroma profile of produces, such as fermented cocoa beans using machine learning modeling from aerial imagery, automatic identification of individual plants and applying equations 1–6 to each sub-image [14].

Water deficit strategies can be used to control the canopy vigor and modify the microclimate in grapevines [33,34] to delay lower-canopy vigor close to harvest, which is consistent with the start of the senescence process [35]. There is an evident effect of canopy architecture and vigor on the microclimate, berry composition, and flavour and aroma development. The machine-learning model based on canopy architecture carried out in this study (Model 2; Figure 5b), with its high accuracy (Table 3) in the estimation of both LT and BCD, could mean that imagery collected using smartphone applications, such as the VitiCanopy App, might be used to predict the levels of berry cell death development in berries [36]. Further machine learning models based on BCD and LT could relate these parameters to berry and wine quality traits, such as aroma profiles [3], achieved in Models 3 and 4 (Figure 6a,b).

The main advantage of this model based on canopy architecture is that historical high resolution (e.g., satellite data) available from a specific wine region could be used to apply the model and obtain wine quality trait targets from available wine libraries [37]. Many wineries preserve a library of their wines from different vintages (vertical vintages), which can be used for chemical and sensory analysis to obtain targets for machine learning validation in other wine regions.

## 5. Conclusions

The implementation of AI in viticulture and winemaking offers powerful tools to assess berry quality traits, such as potential aroma profiles of wines based on berry cell death. Canopy architecture parameters used as ML inputs can be easily acquired through cover imagery or unmanned aerial vehicles (UAV), making the applications and models presented in this paper very practical to assess the spatial and temporal variability of quality traits. Furthermore, the canopy architecture model (Model 2) showed higher accuracy and better performance to Model 1 produced using NIR spectroscopy to predict LT and BCD. However, NIR instrumentation within the range required for the models can be economically prohibitive for small and medium vineyards, requiring know-how for the use, download and management of data. In the case of canopy architecture inputs, they have been already automated and can be easily incorporated as inputs to ML models developed. These models can be used as objective maturity assessments of berries in the field using precision viticulture tools and potentially assess wine quality traits if specific winemaking processes are incorporated within the ML procedure. Further research is required to model other cultivars and winemaking techniques. The results from this research can be incorporated into the models developed here by using the learning capabilities of ANN modelling techniques to acquire a universal model applicable to different regions and cultivars. Specific deployments of machine learning models developed should be determined according to the wine style from different wine producers and feed new relevant data into the models for retraining purposes. The latter procedure will not require an entirely new experiment but enough site-specific data for models to learn the variations required.

## Figures and Tables

**Table 1:** Descriptors presented in the descriptive sensory questionnaire for wine samples of Chardonnay and Shiraz cultivars.

Descriptor	Label	Anchors
Chardonnay		
Clarity	Clarity	Turbid-Brilliant
Color	Color	Colorless-Green-yellow-Yellow-Golden-brown
Aroma Floral	AFloral	Absent-Intense
Aroma Citrus	ACitrus	Absent-Intense
Aroma Spicy	ASpicy	Absent-Intense
Aroma Oak	AOak	Absent-Intense
Aroma Smoke	ASmoke	Absent-Intense
Aroma Sweet	ASweet	Absent-Intense
Aroma Cut Hay	ACut Hay	Absent-Intense
Overall Quality	OQuality	Unacceptable-Extraordinary
Shiraz		
Clarity	Clarity	Turbid-Brilliant
Color	Color	Purple-Ruby-Garnet-Tawny
Aroma Floral	AFloral	Absent-Intense
Aroma Red Fruits	ARedFruits	Absent-Intense
Aroma Black Fruits	ABlackFruits	Absent-Intense
Aroma Sweet	ASweet	Absent-Intense
Aroma Pepper	APepper	Absent-Intense
Aroma Oak	AOak	Absent-Intense
Aroma Mushrooms	AMushrooms	Absent-Intense
Overall Quality	OQuality	Unacceptable-Extraordinary

**Table 2:** Means (top values) and standard error (bottom values) of canopy architecture parameters of the three groups of samples of Chardonnay and Shiraz.

	LAI	Canopy Cover (f_c_)	Crown Cover (f_f_)	Crown Porosity (ϕ)	Clumping Index (Ω)
Chardonnay ^NS^					
Group 1	2.13	0.67	0.80	0.16	0.75
	±0.06	±0.01	±0.02	±0.01	±0.02
Group 2	1.99	0.64	0.78	0.18	0.76
	±0.24	±0.04	±0.05	±0.03	±0.05
Group 3	1.93	0.66	0.81	0.19	0.80
	±0.12	±0.03	±0.04	±0.01	±0.03
Shiraz					
Group 1	1.27 ^b^	0.49 ^b^	0.67 ^b^	0.28 ^a^	0.78 NS
	±0.17	±0.05	±0.06	±0.02	±0.02
Group 2	1.61 ^ab^	0.57 ^ab^	0.72 ^ab^	0.21 ^a^	0.75
	±0.07	±0.02	±0.02	±0.01	±0.02
Group 3	2.00 ^a^	0.68 ^a^	0.84 ^a^	0.19 ^b^	0.82
	±0.07	±0.02	±0.02	±0.01	±0.02

Different letters ^a,b^ represents significant differences between groups based on ANOVA (*p* < 0.05) and Tukey post hoc test (α = 0.05). ^NS^: non-significant differences.

**Table 3:** Statistical data for each stage of the artificial neural network regression Models 1 and 2.

Stage	Samples	Observations	R	Slope	Performance (MSE)
Model 1: inputs: near-infrared absorbance; targets: living and dead tissue					
Training	324	648	0.91	0.82	35.2
Testing	108	216	0.77	0.81	106.5
Overall	432	864	0.87	0.82	-
Model 2: inputs: canopy architecture; targets: living and dead tissue					
Training	260	520	0.98	0.95	8.9
Validation	86	172	0.98	0.91	11.5
Testing	86	172	0.98	0.91	11.4
Overall	432	864	0.98	0.93	-

Abbreviations: R: correlation coefficient; MSE: means squared error.

**Table 4:** Statistical data for each stage of the artificial neural network regression Models 3 and 4 developed using canopy architecture parameters as inputs to predict the intensity of sensory descriptors.

Stage	Samples	Observations	R	Slope	Performance (MSE)
Model 3: Chardonnay					
Training	130	1300	0.99	0.99	0.04
Validation	43	430	0.99	0.99	0.06
Testing	43	430	0.99	0.99	0.06
Overall	216	2160	0.99	0.99	-
Model 4: Shiraz					
Training	130	1300	0.99	0.99	0.03
Validation	43	430	0.99	1.00	0.05
Testing	43	430	0.99	1.00	0.05
Overall	216	2160	0.99	1.00	-

Abbreviations: R: correlation coefficient; MSE: means squared error.

## Data Availability

Data and intellectual property belong to The University of Melbourne; any sharing needs to be evaluated and approved by the University.

## Supplementary Materials

The following are available online at https://www.mdpi.com/article/10.3390/s21217312/s1: Figure S1. Diagram depicting the methods to obtain inputs to feed the proposed models to obtain the specific outputs; Figure S2. Means of living tissue and effective leaf area index of the three groups of samples of Chardonnay (green) and Shiraz (red) at harvest time. Error bars depict the standard error. Different letters represent significant differences between groups based on ANOVA (*p* < 0.05) and Tukey post hoc test (α = 0.05); Table S1. Means (top values) and standard errors (bottom values) of sensory descriptors of the three groups of samples of Chardonnay and Shiraz wines.
