# Supplementary material for: Berry Cell Vitality Assessment and the Effect on Wine Sensory Traits Based on Chemical Fingerprinting, Canopy Architecture and Machine Learning Modelling
Source: Sensors (Basel). 2021 Nov 3;21(21):7312. doi: 10.3390/s21217312 (PMC8587162; doi:10.3390/s21217312)
Supplement: Supplementary file 1 [file sensors-21-07312-s001.zip › sensors-1423826-supplementary.pdf]

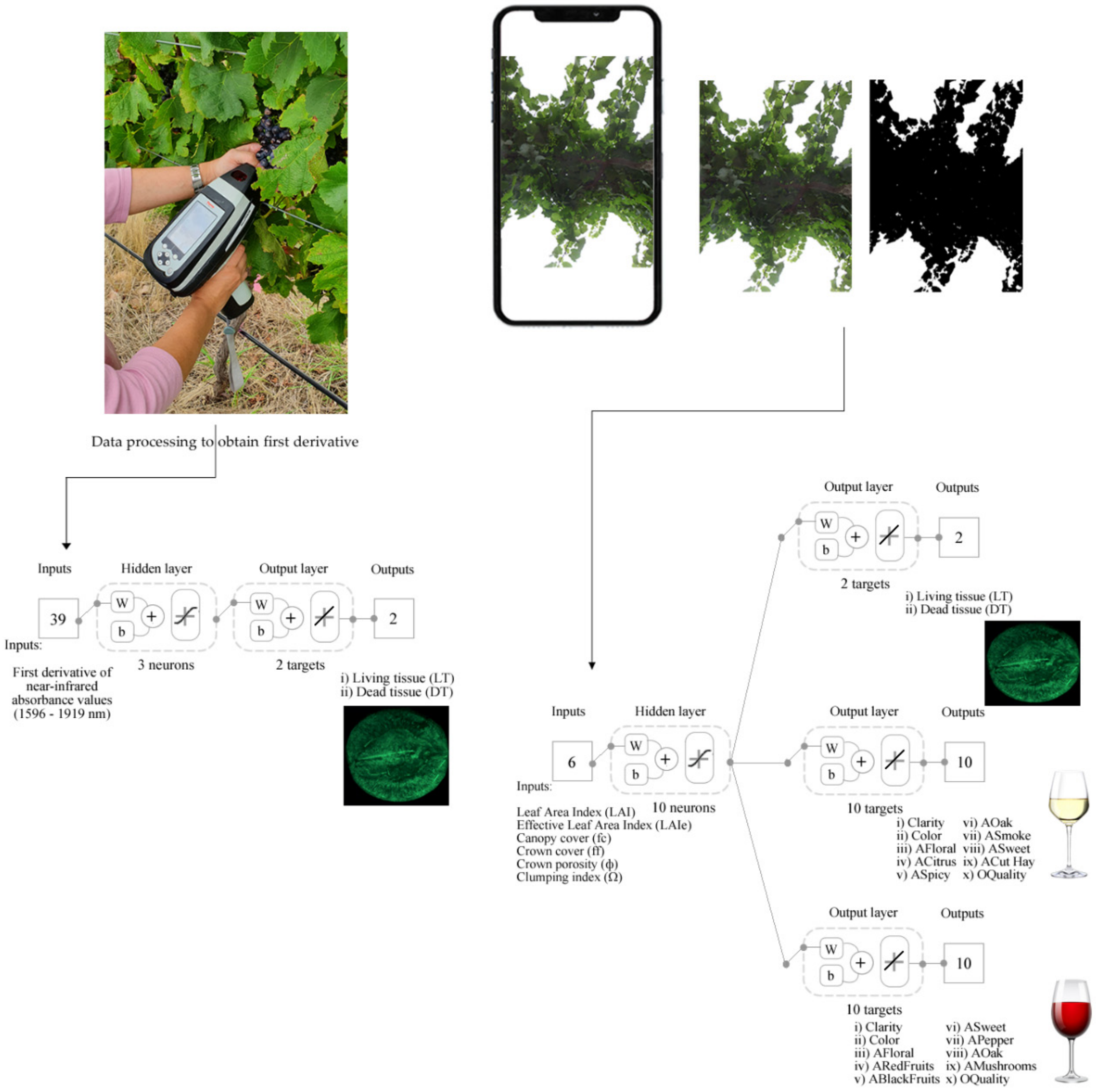

Figure S1. Diagram depicting the methods to obtain inputs to feed the proposed models to obtain the specific outputs.

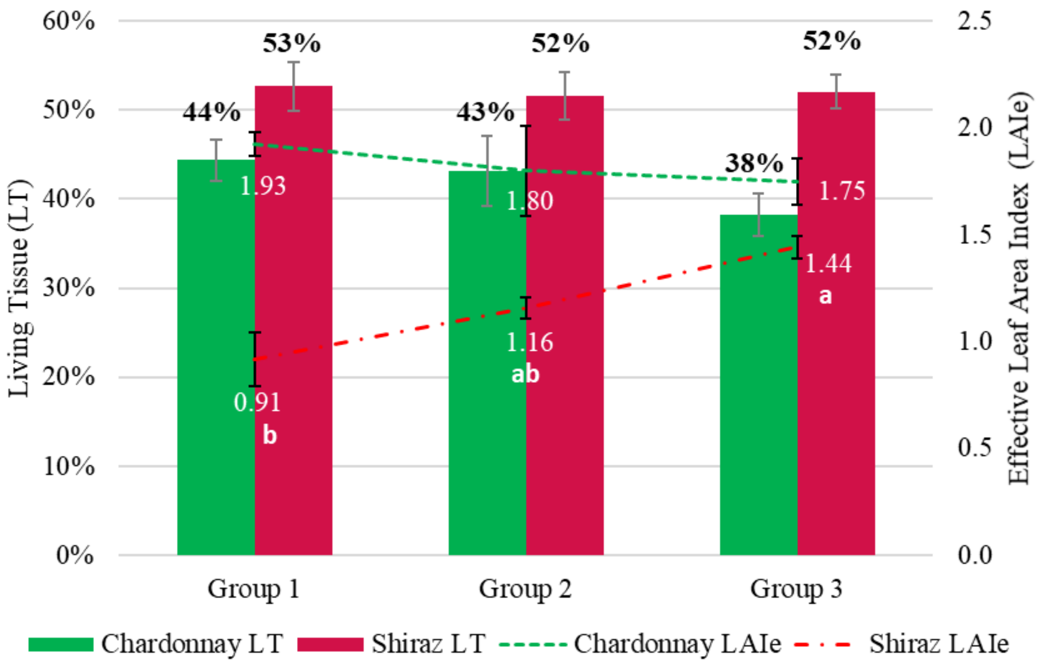

Figure S2. Means of living tissue and effective leaf area index of the three groups of samples of Chardonnay (green) and Shiraz (red) at harvest time. Error bars depict the standard error. Different letters [ab] represents significant differences between groups based on ANOVA ($p < 0.05$) and Tukey post hoc test ($\alpha = 0.05$).

Table S1. Means (top values) and standard error (bottom values) of sensory descriptors of the three groups of samples of Chardonnay and Shiraz wines.

Sample/Attribute	Clarity	Color	Aroma Floral	Aroma Citrus	Aroma Spicy	Aroma Oak	Aroma Smoke	Aroma Sweet	Aroma Cut Hay	Overall quality
					**Chardonnay**					
**Wine Group 1**	4.88<br>±1.22	4.93<br>±1.05	5.23<br>±1.32	5.64<br>±1.30	2.36<br>±1.04	3.71<br>±0.75	2.45<br>±1.19	5.95<br>±1.65	6.25<br>±1.03	9.90<br>±0.43
**Wine Group 2**	3.77<br>±1.25	5.92<br>±0.87	6.33<br>±1.10	7.11<br>±1.07	3.24<br>±0.81	2.89<br>±0.88	2.77<br>±0.76	6.86<br>±1.60	6.43<br>±1.25	9.66<br>±0.54
**Wine Group 3**	4.89<br>±1.20	3.3<br>±1.23	8.04<br>±0.97	6.15<br>±1.45	2.57<br>±0.77	2.25<br>±1.24	1.58<br>±0.81	7.73<br>±1.45	4.61<br>±0.96	10.58<br>±0.39
**Sample/Attribute**	**Clarity**	**Color**	**Aroma Floral**	**Aroma Red Fruits**	**Aroma Black Fruits**	**Aroma Sweet**	**Aroma Pepper**	**Aroma Oak**	**Aroma Mushrooms**	**Overall quality**
					**Shiraz**					
**Wine Group 1**	5.31<br>±0.88	5.36<br>±0.84	5.33<br>±1.35	7.27<br>±1.2	6.71<br>±1.23	7.96<br>±1.48	2.56<br>±0.94	3.37<br>±1.45	4.91<br>±1.17	10.20<br>±0.57
**Wine Group 2**	8.04<br>±1.05	6.80<br>±0.88	4.42<br>±1.01	7.33<br>±1.02	6.51<br>±1.42	7.56<br>±1.43	1.89<br>±0.37	4.85<br>±1.26	5.39<br>±1.16	10.90<br>±0.40
**Wine Group 3**	6.49<br>±1.03	6.65<br>±0.66	5.21<br>±1.32	8.38<br>±1.19	5.45<br>±0.82	5.72<br>±1.54	2.97<br>±1.16	4.03<br>±1.31	5.75<br>±1.49	10.72<br>±0.50
